# The Epibiotic Microbiota of Wild Caribbean Sea Urchin Spines Is Species Specific

**DOI:** 10.3390/microorganisms11020391

**Published:** 2023-02-03

**Authors:** Ruber Rodríguez-Barreras, Anelisse Dominicci-Maura, Eduardo L. Tosado-Rodríguez, Filipa Godoy-Vitorino

**Affiliations:** 1 Department of Biology, University of Puerto Rico, Mayagüez Campus, P.O. Box 9000, Mayagüez 00681-9000, Puerto Rico; 2 Department of Microbiology, University of Puerto Rico School of Medicine, Guillermo Arbona Main Building, San Juan 00936-5067, Puerto Rico

**Keywords:** sea urchin, epibiotic microbiome, 16S rRNA, Caribbean, Puerto Rico

## Abstract

Caribbean sea urchins are marine invertebrates that have experienced a decline over the years. Studies on sea urchins have focused primarily on the microbiome of the coelomic fluid or the gut microbiota. In this study, the epibiota community associated with four wild Caribbean sea urchin species, *Lytechinus variegatus*, *Echinometra lucunter*, *Tripneustes ventricosus*, and *Diadema antillarum*, was characterized for the first time. Using 57 sea urchin animal samples, we evaluated the influence of animal species, trophic niches, and geographical location on the composition of the epibiotic microbiota. We found significant differences in the bacterial biota among species and trophic niches, but not among geographical locations. *L. variegatus* exhibited the highest alpha diversity with high dominance of Fusobacteria, Planctomycetes, and Cyanobacteria, whereas *T. ventricosus* and *D. antillarum* were dominated by Firmicutes. *T. ventricosus* inhabiting the seagrass biotope dominated by *Thalassia testudinum* meadows had mostly *Endozoicomonas.* In contrast, samples located in the reef (dominated by corals and other reef builders) had a higher abundance of *Kistimonas* and *Photobacterium.* Our findings confirm that the epibiotic microbiota is species-specific, but also niche-dependent, revealing the trophic networks emerging from the organic matter being recycled in the seagrass and reef niches. As echinoids are important grazers of benthic communities, their microbiota will likely influence ecosystem processes.

## 1. Introduction

Over the last two decades, microbiological studies have advanced from traditional cultures and microscopy to sophisticated genomic and molecular analyses [1,2]. The developments of refined genomic sequencing and bioinformatic tools have revolutionized the traditional microbiology approaches, exponentially increasing the existing knowledge of prokaryotes and host microbiomes [3,4]. Several studies using high-throughput 16S rRNA gene sequencing have been published in vertebrates such as fish, birds, and mammals [5,6,7], while marine invertebrates have received less attention [8,9,10]. In animals, the external surface, whether the skin, carapace, or spines (tests), is considered the primary physical barrier between the organism and its surrounding environment. This superficial layer is colonized and inhabited by a diverse collection of microorganisms including viruses, archaea, bacteria, fungi, and micro invertebrates [10,11]. These microbial communities not only evolve with the host but also vary due to age, diet, anatomical region, and other physicochemical factors [12,13,14,15]. A diversity profile of external microbiota has been previously described in animals, most of them focused on vertebrates, such as domesticated animals and amphibians [16,17,18], with a few studies in reptiles and fish [11,19]. The external microbiota of invertebrates remains poorly studied with few published works in anthozoans and echinoderms [1,20,21].

Echinoderms are a group of more than 7000 living species of invertebrates classified into five Classes [22,23]. In Puerto Rico, 19 species of the 108 shallow-water echinoderms belonging to the Class Echinoidea can be found (sea urchins, sand dollars, and sea biscuits) [24]. Their occurrence in coral reefs and other shallow-water ecosystems is important [25] because they protect young fish recruits and alter substrate properties through sediment bioturbation [26,27]. Sea urchins inhabit all marine ecosystems, from coastal to abyssal, and from the tropics to the poles at all depths, in a variety of biotopes, from the intertidal zone to the abyssal regions [28]. They are also considered important grazers with a strong ecological impact in benthic communities [29,30] and serve as excellent indicators of the health of marine ecosystems due to their biological significance and abundance in benthic environments [31]. Moreover, sea urchins are marine models that have been used extensively for scientific investigations in ecology, toxicology, aquaculture, development, genetics, and many other fields [32,33,34,35,36].

Four of the most distinct sea urchin species found in Puerto Rico are *Diadema antillarum*, *Lytechinus variegatus*, *Echinometra lucunter*, and *Tripneustes ventricosus*. *D. antillarum* and *E. lucunter* are inhabitants of coral reefs and are often associated with the hardground biotope, where they feed primarily on macroalgae, but also small invertebrates. Furthermore, *L. variegatus* and *T. ventricosus* are usually inhabitants of the back-reef biotope dominated by seagrass meadows of *Thalassia testudinum*. These omnivorous echinoids forage on *T. testudinum* leaves, but also on macroalgae and small invertebrates [37,38].

The body surface of sea urchin species also contains epibiotic symbionts. Learning about the epibiotic composition is an important step towards understanding the normal microbiota and eventually understanding changes caused by diseases [39]. One of the studied species, *D. antillarum*, suffered a mass mortality event in the 1980s, caused by an unknown pathogen [38,40]. The lack of this keystone grazer promoted uncontrolled algal overgrowth, affecting reef-building anthozoans [41], with a consequent collapse of coral reefs. This die-off and another recent one in 2022 [42] mark the importance of studying the microbial community linked to these grazers. The external microbiome has been linked with adaptive evolution and host immunity with important implications for host well-being and fitness [43,44]. However, the importance of external microbiota, even though critical for immunity response, has been understudied in invertebrates [10,45,46]. Microbiome studies in echinoderms have been focused primarily on the coelomic fluid and gut microbiota [4,47,48], whereas the external microbiota remains unstudied.

We aimed to understand the composition of epibiotic bacteria inhabiting the spines of these echinoids, as well as their external microbiota, in different biotopes to gain insight into how local conditions could affect microbial assemblages. This study represents one of the first efforts to characterize bacterial taxonomic differences associated with wild-caught Caribbean sea urchin species and compare these microbial communities between trophic niches and geographical locations.

## 2. Materials and Methods

### 2.1. Study Site and Sample Collection

Surveys were carried out in February 2019 at three shallow-water sites (1–2 m depth) along Puerto Rico’s northeastern coast. Cerro Gordo in Vega Baja 18°29′06.0″ N; 66°20′20.1″ W), Isla de Cabra in Cataño (18°28′26.6″ N; 66°08′18.5″ W), and Punta Bandera in Luquillo 18°23′16.0″ N; 65°43′05.2″ W) were chosen for the study. At each location, physical data were taken. A quality meter Pro 2030 (https://www.ysi.com/pro2030, accessed on 10 December 2019) was used to measure water temperature, salinity, and pH. More information about the site can be found in Rodríguez-Barreras et al. [4]. Temperature, salinity, and pH were similar among sites, ranging from 25.6–26.8 °C, 33.2–33.8, and 8.33–8.40, respectively (Supplementary Table S1).

At each of the three sites, six adults of the species *D. antillarum*, *E. lucunter*, and *T. ventricosus* were collected. In addition, three adults of *L. variegatus* were collected in CG and IC, but no *L. variegatus* individuals were identified in MA. To note, *T. ventricosus* individuals were collected close to the border between the seagrass and the reef biotopes and *L. variegatus* was rare to find and the individuals collected were far from the reef sea urchins. The gut microbiota, seagrass, and water analyses for these same individuals, for which the spine microbiota was studied in this paper, had been previously published [4].

We collected a total of 60 animals. *E. lucunter* and *D. antillarum* were found on hardground biotopes (fringing reefs), whereas *L. variegatus* and *T. ventricosus* were found in a seagrass biotope dominated mostly by *Thalassia testudinum*. Sea urchins and seagrass were isolated in separate plastic bags containing seawater. All samples were temporarily preserved in a foam cooler before being promptly transported to the laboratory for processing. The Department of Natural and Environmental Resources of Puerto Rico approved this study (permit # DRNA-2019-IC-003). The University of Puerto Rico Medical Sciences Campus IACUC #A5301118 approved the animal dissection protocol.

### 2.2. Sample Processing

Specimens were conditioned in seawater for at least 10 min in 100 mL glass beakers with 25 mL saltwater, or until they stuck to the beaker surface, according to the authorized IACUC protocol A5301118. Once attached, 25 mL of a sterile solution of 20 mM MgCl2 was added for sedation as per the IACUC protocol. This is an anesthetic routinely used in aquaculture research [49]. After standardized exposure of 15 min, experimentally induced anesthesia was observed until all participants disengaged from the walls of the beaker. Animals were carefully relocated by hand into a metal tray and placed in ultra-low temperatures (−80 °C) for 10 min before dissection.

Sea urchins were placed in a natural position with the oral surface facing the metal tray. Using flame-sterilized scissors, spines were cut around the base and removed. The endoskeleton was meticulously dissected with a scissor and a 2 cm × 2 cm square section at the equatorial line was cut. The exterior fragment (used in this study), including test fragments and proximal spines, was gently removed using tweezers to avoid contamination with the internal content and fluids, then transferred to 2 mL microtubes and frozen at −80 °C until DNA extraction. Additionally, gut content samples (primarily pellets with a few fragments of intestinal tissue) were also collected for a biorepository [4].

### 2.3. Genomic DNA Extractions

Each ~200 mg of sea urchin species’ tests (proximal spines) was used for DNA extraction with the QIAGEN PowerSoil kit (QIAGEN LLC, Germantown Road, MD, USA). The protocol was slightly modified for complete homogenization of the spine samples using a first step at 3000 r.p.m. for 2 min at room temperature, with the PowerLyzer (QIAGEN LLC, Germantown, MD, USA). The last elution step used 100 µL of sterile PCR water previously warmed at 65 °C, which remained on the filter for 5 min at room temperature prior to the final centrifugation step. All the other extraction steps were performed following the standard protocol. DNA was measured using the Qubit^®^ dsDNA HS (High Sensitivity) assay kit (Waltham, MA, USA).

### 2.4. 16S rRNA Gene Amplification, Sequencing, and QC Processing

The l6S library preparation was performed at the sequencing facility following the amplification protocol of the Earth Microbiome Project [50] using the V4 hypervariable region of the 16S ribosomal RNA marker gene (291 bp) as previously described [4]. After PCR, amplicons were quantified and volumes of each of the products were pooled into a single tube so that each amplicon was represented in equimolar amounts. This pool was then cleaned using AMPure XP Beads (Beckman Coulter) and quantified using a fluorometer (Qubit, Invitrogen, Waltham, MA, USA). After quantification, the molarity of the pool was determined. It diluted down to 2 nM, denatured, and then diluted to a final concentration of 6.75 pM with a 10% PhiX spine for sequencing on the Illumina MiSeq. The sequencing facility ran negative controls and sequenced our gDNA extraction negative controls sent along with samples. Nothing is reported if they did not produce sequence reads above 500 total reads. The positive control analyzed and aligned to the sequencer in real-time would be shared only if a run had an error rating above 1%, but this was not the case. QIITA was used to deposit the raw 16S-rRNA reads and associated metadata [51]. The raw sequences are available in the European Nucleotide Archive ENA Project: PRJEB40117 and ERP123720, and in bioproject ID 12668. Pre-processing of demultiplexed files was performed with a Phred offset parameter of 33 and default values using split libraries FASTQ (QIIMEq2 1.9.1) [52]. Reads were trimmed to 250 bp and the reference database was SILVA [53] with a closed-reference OTU picking method, using a minimum similarity threshold of 97%.

### 2.5. Bioinformatic Analyses and Statistical Tests

The species table (biom file), which was obtained from QIITA [52], was used for downstream analyses with a locally operated version of QIIME2 [54]. We removed OTUs with fewer than five reads, chloroplast and mitochondrial-like sequences, several matches with eukaryotes, and taxonomically unassigned sequences for downstream analyses. We selected a rarefaction level of 2800 reads for a total of 50 samples that did not include *Lytechinus variegatus* (green) because the rarefaction threshold for analyses that included *L. variegatus* (n = 57) was 1300 reads [1,2]. The chosen rarefaction level resulted in the removal of 3 samples.

### 2.6. Beta Diversity Analyses

Pairwise Bray–Curtis dissimilarity distances between samples were computed for community-level analyses. Non-metric multidimensional scaling (NMDS) [55] was used to show global differences in bacterial community composition and structure utilizing collection sites, habitats, and sea urchin species as metadata categories. ANOSIM tests were used for statistical significance assessment between sample groups using a non-parametric statistical test that compares ranking beta diversity distances between different group depths [56]. Additionally, PERMDISP was used to determine whether the dispersions between the groups were significantly separated. These tests were executed in QIIME2 using the script qiime diversity beta-group-significance, with the distance matrix as the input file and 999 permutations.

### 2.7. Alpha Diversity, Taxonomic Barplots, and LEfSe Analysis

The observed species (OTU present in the sample), Chao 1 index (richness) [57], Shannon (diversity) [55], and Evenness (bacterial distribution in the sample) [58] values were shown as boxplots using R [56]. We utilized the script “qiime diversity alpha-group-significance” in QIIME2 to compare the alpha diversity between groups of samples in each metadata category. Nonparametric t-tests using Monte Carlo permutations were used to obtain the p-value in these statistical tests. MicrobiomeAnalyst [59] was used to generate bar plots indicating phylum and genus level taxa, as well as linear discriminant analysis (LDA) effect size (LefSe) [60,61]. We did not normalize, scale, or apply filters in this web platform because the data were already normalized using QIIME2. We utilized relative abundance (%) to illustrate taxonomic distribution [62]. LefSe was calculated to determine enrichment in the categories of interest given the taxonomic profiles. Taxa with LDA scores greater than two at a p-value of 0.05 were considered statistically significant for both species and trophic niches. R was used to generate a list of shared core prevalent taxa To identify the most prevalent taxa in each category, we used filtering parameters: 0.0001 for detection (taxa in at least 90% of samples) and 0.1 for prevalence. This resulted in a reduced list of OTUs used to generate Venn diagrams of shared core taxa. The obtained list of OTUs was entered into a web-based Venn diagram tool plotted with InteractiVenn [63,64].

## 3. Results

### 3.1. General Description of Animal Samples

A total of 2,245,845 of 16S rRNA raw reads were obtained for the 57 samples, but 1,380,601 good-quality sequence reads and 15,474 OTUs remained after trimming and quality assessment. Spatial comparisons found that Cataño was the site with the highest number of reads and OTUs, whereas Luquillo (MA) was the least diverse site with 326,330 reads and 3774 OTUs (Table 1). Among species by site, in Cataño, the external microbiota of *E. lucunter* exhibited the highest number of reads and OTUs, producing 243,333 reads and 2645 OTUs, followed by *T. ventricosus* and *D. antillarum*; the last position was occupied by *L. variegatus* producing 10,116 reads and 844 OTUs. The species *T. ventricosus* in Cerro Gordo and *D. antillarum* in Luquillo exhibited the lowest number of reads and OTUs among the three collecting sites (Table 1).

The overall number of reads and OTUs varied by species. The highest number of reads was obtained in *E. lucunter* with 532,034 reads, followed by *T. vetricosus*, while *L. variegatus* exhibited the lowest number of reads with 11,537 reads due to a low number of available samples (Table 2). A similar result was obtained with the number of OTUs, where *E. lucunter* displayed the highest number of OTUs and *L. variegatus* the species with the lowest number. *D. antillarum* and *T. ventricosus* exhibited a similar number of OTUs.

### 3.2. Comparisons of Epibiotic Microbiota between Sea Urchin Species

**Figure 1:**
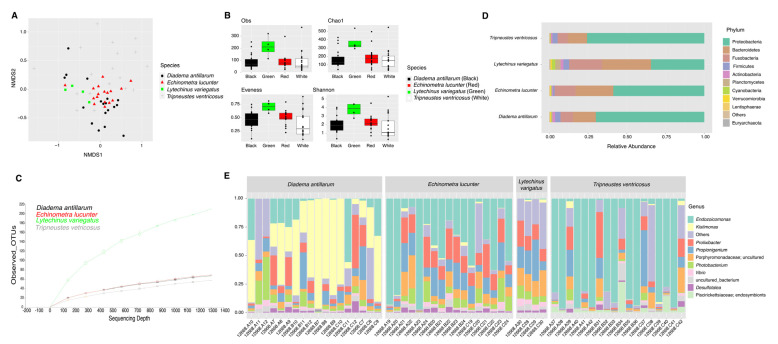
Bacterial composition and diversity of four sea urchin species. Bray–Curtis analysis, represented by an NMDS (stress = 0.212), using species as metadata categories, depicts distinct species clustering with ANOSIM p_val_ = 0.001 (**A**). Alpha diversity analyses revealed significant differences (*p* value < 0.05) in alpha diversity (Shannon) and richness (Chao1 and Observed species) analyses between species when compared to *L. variegatus* (green sea urchin) (**B**). Panel (**C**) depicts rarefaction curves at the species level. Species-relative abundance of top ten taxa at phyla (**D**) and genus levels (**E**) are depicted by the barplots. For significant p-values refer to Supplementary Table S3.

Community structure analysis displayed significant differences among sea urchin species (Supplementary Table S2). *D. antillarum* composition significantly differed from those observed in *L. variegatus* (ANOSIM, p_val_ = 0.008)*, T. ventricosus* (ANOSIM, p_val_ = 0.001), and *E. lucunter* (ANOSIM, p_val_ = 0.001) (Figure 1A). *E. lucunter* community structure was found to be significantly different from those observed on *L. variegatus* (ANOSIM, p_val_ = 0.016) and *T. ventricosus* (ANOSIM, p_val_ = 0.001) (Figure 1A). Furthermore, *L. variegatus* composition differed from that observed in *T. ventricosus* (ANOSIM, p_val_ = 0.001) (Figure 1A). Nevertheless, PERMDISP results showed no significant differences among species (overall, p_val_ = 0.728) (Supplementary Table S2).

External microbiota of *L. variegatus* were more diverse (Shannon diversity index) with respect to *E. lucunter* (*t*-test, p_val_ = 0.015), *D. antillarum* (*t*-test, p_val_ = 0.008) and *T. ventricosus* (*t*-test, p_val_ = 0.022) (Figure 1B) (Supplementary Table S3). Richness analysis (observed species and Chao1) revealed the same trend as Shannon, with *L. variegatus* showing significantly more abundance when compared to *E. lucunter* (*t*-test, obs. species p_val_ = 0.011; Chao1 p_val_ = 0.012), *D. antillarum* (*t*-test, obs. species p_val_ = 0.017; Chao1 p_val_ = 0.021), and *T. ventricosus* (*t*-test, obs. species p_val_ = 0.017; Chao1 p_val_ = 0.006) (Figure 1B) (Supplementary Table S3). Evenness revealed a different trend with differences between *E. lucunter* and *D. antillarum* (*t*-test, evenness p_val_ = 0.021), and *E. lucunter* and *T. ventricosus* (*t*-test, evenness p_val_ = 0.006) (Figure 1B) (Supplementary Table S3).

The relative abundance of the external microbial community at the phylum level displayed a higher dominance of Fusobacteria, Cyanobacteria, and Planctomycetes in *L. variegatus animals* compared to the other species. *T. ventricosus* (white) *and D. antillarum* were proportionately higher by Firmicutes. A similar abundance of Bacteroidetes, Proteobacteria, and Fusobacteria was found among all sea urchins (Figure 1C). *Prolixibacter, Photobacterium*, and *Propionigenium* were the most abundant bacterial genera, but some samples of *T. ventricosus* and *E. lucunter* displayed a higher dominance of *Endozoicomonas* (Proteobacteria), and *Kistimonas* (Proteobacteria) were particularly abundant in *D. antillarum* samples (Figure 1D).

**Figure 2:**
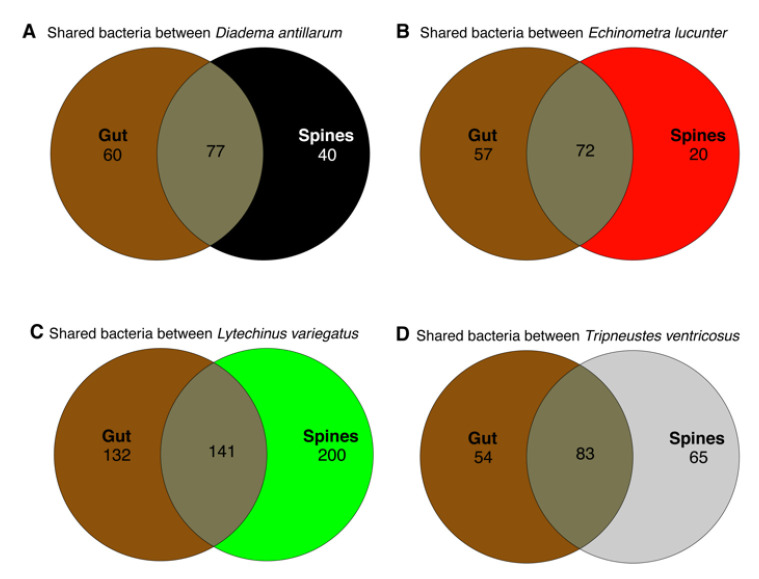
Venn diagram modeling shared and unique genus-level taxa considering core taxa with detection of 0.001 among the four sea urchin species in the gut and spine samples. Gut sample data used in this analysis were previously published [4].

We wanted to understand the number of bacteria shared by the animal surface and their gut. We computed core taxa shared between gut and spines modeled through Venn diagrams to reflect the amount of exclusive and shared bacterial species between body sites by each sea urchin species. The highest number of unique and shared species was found in *L. variegatus*. In general, unique species were higher in gut samples, whereas the external community exhibited fewer unique bacterial species. In contrast, *L. variegatus* exhibited an inverse result with 200 species in the surface samples vs. 132 in the gut samples (Figure 2). A list of identified taxa as unique for the test or shared with the gut is available (Supplementary Table S4).

### 3.3. Comparison of the Microbiota between Reef and Seagrass Niches

**Figure 3:**
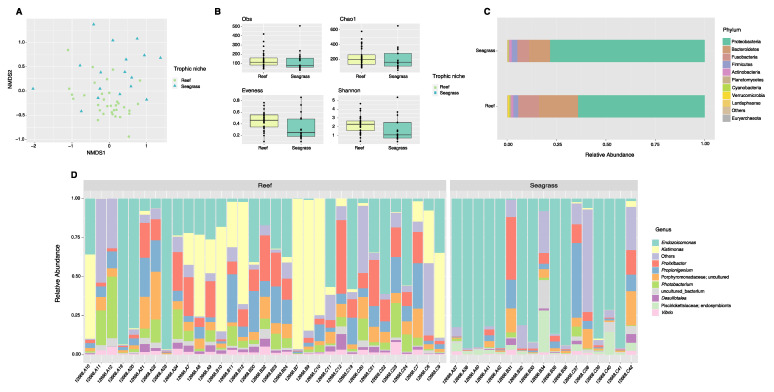
Diversity analyses comparing spine microbiota of sea urchin trophic niches (animals collected in the reef or among seagrass). Bray–Curtis analysis, represented by an NMDS (stress 0.1943077), ANOSIM *p*-value = 0.001 (**A**). Alpha diversity exhibits significant differences (p_val_ < 0.05) in alpha diversity (Shannon) and evenness between trophic niches (**B**). Species-relative abundance of top ten taxa at phyla (**C**) and genus levels (**D**) are depicted by the barplots. Refer to Supplementary Table S3 for significant *p*-values.

Community analyses by animal niche (biotope) showed significant differences between the reef and seagrass bacterial compositions (ANOSIM, p_val_ = 0.001, Figure 3A, Supplementary Table S2). Although alpha diversity analyses exhibited differences between sample biotopes (Figure 3B, Supplementary Table S3), these cannot be separated from the species effect. Indeed, *Diadema* and *Echinometra* (species with certain genus-level similarities) both inhabit the reef. In contrast, *Tripneustes and Lytechinus* inhabit the seagrass biotope (dispersion not significant), likely given that *Tripneustes* samples were collected bordering the reef. In terms of phyla, broadly, Fusobacteria and Verrucomicrobia dominated reef samples while seagrass samples were dominated by Proteobacteria and showed reduced *Fusobacteria* (Figure 3C). At the genus level, reef samples were dominated by *Kistimonas* and *Photobacterium* while seagrass samples were dominated by *Arenicella* (Figure 3D).

When combining both species and their biotopes, data show that the epibiotic microbes inhabiting reef species cluster together, as compared to those in *T. ventricosus* (seagrass) (Figure 3E).

### 3.4. Putative Biomarker Identification Using Linear Discriminant Analysis Effect Size (LEfSe)

**Figure 4:**
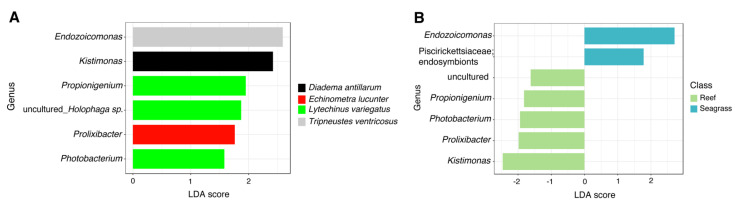
Differences in relative abundances of genus-level taxa between (**A**) sea urchin species and (**B**) trophic niches (reef vs. seagrass) by LEfSe analysis (Logarithmic LDA score > 2.0; alpha value < 0.05).

LefSe analysis revealed significantly more abundant bacterial genera across sea urchin species. *L. variegatus* exhibited a high abundance of *Propionigenium, Photobacterium,* and an uncultured taxon, making it the sea urchin with more significantly different taxa. The other three species of sea urchins exhibited only one significant genus per sea urchin with *Ecdizoicomonas* in *T. ventricosus*, *Kistimonas* in *D. antillarum*, and *Prolixibacter* in *E. lucunter* (Figure 4A). Sea urchins collected from seagrass biotopes displayed a greater abundance in *Endozoicomonas* and endosymbionts, whereas sea urchin samples from the reef biotope displayed lesser abundance in five taxa including the genera *Propionigenium*, *Photobacterium*, *Prolixibacter*, *Kistimonas,* and an uncultured bacterium (Figure 4B).

### 3.5. Comparing Sea Urchin Microbiota among Geographical Locations

**Figure 5:**
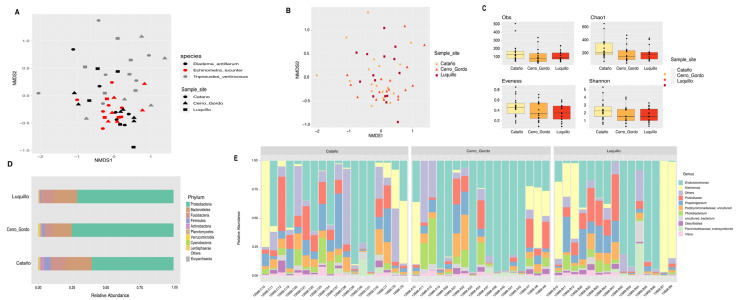
Spatial diversity analyses of spine microbiota comparing animals collected in Cataño, Cerro Gordo, and Luquillo. NMDS plot depicting a combination of species and sample locations shows that differences are due to the species at each location (**A**); for p-values, see supplementary Table S2. Bray–Curtis analysis, represented by an NMDS (stress = 0.1943), used sites and sea urchin species as metadata categories. ANOSIM shows no significant differences between sample sites (**B**). No alpha diversity estimates show any significant differences between sample sites (**C**). Species relative abundance of top ten taxa at phyla (**D**) and genus levels (**E**) are depicted by the bar plots.

Microbial community analyses comparing the epibionts of sea urchins collected at three different sites considered only individuals present in all these sites (that is, we disregarded *L. variegatus* as it did not appear in Luquillo and only one individual was found in Cerro Gordo, as shown in Table 1 and Table 2). Most *Diadema* and *Echinometra* samples have similar external microbiota regardless of sampling sites as these are both reef-belonging species. However, the dispersion in *Tripneustes* individuals was not significantly different compared to other species (Figure 5A, Supplementary Table S2). Alpha diversity analyses showed that Cataño exhibited higher alpha diversity, which was, nonetheless, only moderately significant in the observed species (*p* = 0.055, Supplementary Table S3). The only significant alpha differences we found among sites correspond to evenness between Cerro Gordo and Luquillo (*p* = 0.043, Supplementary Table S3), as shown in Figure 5B. At the phyla level, we found three dominant groups across all sites: Proteobacteria, Bacteroidetes, and Fusobacteria. Proteobacteria dominated Cerro Gordo samples with a reduction of Fusobacteria. Cataño samples were dominated by Actinobacteria and Firmicutes (Figure 5C). At the genus level, we found that *Endozoicomonas* (Proteobacteria), *Kistimonas* (Proteobacteria), and *Prolixibacter* (Bacteroidetes) were found among all samples. The genus *Kistimonas* was dominant in Luquillo, and *Endozoicomonas* was more abundant in Cerro Gordo (Figure 5D). 

## 4. Discussion

The environment and the host’s evolutionary history are considered significant in influencing the interactions between animals and bacteria [65]. We characterized the external bacterial communities (spines) of four Caribbean sea urchin species and compared them according to their species, sampling site, and trophic niche. Characterization of the bacterial microbiota associated with sea urchins has been reported by several studies but is primarily associated with gut and coelomic fluid samples [2,4,9,66], including animals reared in aquaculture [2]. This study is the first of its kind characterizing the external microbiota of four common Caribbean echinoids collected in the wild. These results fill the gap of knowledge that exists on these sea urchin species in the Caribbean.

For instance, approximately 98% of *D. antillarum* perished in the early 1980s due to a waterborne but unidentified disease [40]. Following the removal of *D. antillarum*, reefs suffered a significant rise in macroalgae, which led to a precipitous fall in coral cover over the ensuing decades [40,41]. *D. antillarum* is one of the top grazers of Caribbean coral reefs [67,68]. As a result, annihilating this crucial ecological component could hasten current coral reef deterioration, leading to an unprecedented collapse of Caribbean coral reefs and affecting the associated communities. This makes it essential to investigate the microbiome connected to it in healthy individuals both during and after the outbreak. Recently, there have been reports of *Diadema antillarum* fatalities along the US Virgin Islands reported in February 2022 [42]. Following this first report, numerous reefs in the Caribbean have recorded *D. antillarum* mortalities. Even though infectious illnesses are widespread in the marine environment, mass fatalities are uncommon despite their severe and long-lasting impacts. The data from this study cohort from animals collected in 2019—three years before the current die-off—is hence of utmost importance so that comparisons with animals collected in 2022 allow the identification of taxa lost in the outbreak. It may be crucial in identifying the putative pathogen(s) and potential microbial alterations linked to *D. antillarum* illness.

Our microbiome analysis was used to identify putative core microbiomes commonly shared between the four sea urchin species. Like previous findings with gut samples in the same four species [4], the epibiotic microbiota showed differences in bacterial community structure according to the species and trophic niche. We compared the shared number of species between gut microbiota and the bacteria in the spines of these wild animals, in a similar effort to a study of shallow-water vent crab [69], and preliminarily note that *L variegatus*, a species inhabiting seagrass meadows, has a higher number of species compared to their own gut microbes. Future work could focus on understanding if these external bacteria come from the meadows where they live.

Each species displayed its own external microbial community. Despite that we cannot separate both species and habitat effects, we found the existence of specific epibiotic communities selected according to the animal’s habitat. For example, *T. ventricosus* and *L. variegatus* inhabit the same biotope (Seagrass beds); nonetheless, the external microbiota of *T. ventricosus* was somewhat similar regarding the composition, diversity index (Shannon), and taxonomic distribution at the phyla level to *D. antillarum* and *E. lucunter* from the reef niche. We had previously reported a similar trend for sea urchin gut samples [4]. The species *E. lucunter* and *D. antillarum* inhabit hardgrounds where corals, sponges, and other cnidarians are dominant [22], while *L. variegatus* and *T. ventricosus* usually graze on turtle grass blades (*Thalassia testudinum*) [37]. The unexpected difference in external microbiota between *T. ventriocus* and *L. variegatus*, and the relative similarity between *T. ventricosus* and the two reefs’ species, could be explained somehow due to the migration behavior of this species [70]. Individuals of this species were found and collected close to the limit between the seagrass and back reef zones, at approximately 5 m from the border between the two biotopes. *T. ventricosus* is a common herbivore of seagrass throughout the Caribbean [37]. Although *T. ventricosus* is typically associated with seagrass habitats, it has been observed to migrate to the backreef zone where *D. antillarum* and *E. lucunter* inhabit [70], which could explain their relative similarity in gut bacterial composition [4] and now their epibionts communities too.

Taxonomic profiles at the genus level revealed that Cyanobacteria were more abundant only in *L. variegatus* samples, simultaneous with a dominance of Fusobacteria and Planctomycetes. Cyanobacteria seem to come from the ingestion of *T. testudimun* leaves, which represent one of the most important components in the food chain [24]. *L. variegatus* uses fragments of this seagrass to cover the surface of its body [71], which could explain the high abundance of Cyanobacteria. In contrast, *E. lucunter* and *T*. *ventricosus* exhibited more abundance of *Endozoicomonas* (Proteobacteria), a bacterium frequently found in a variety of marine hosts, including reef-building corals [72]. The sharing of *Endozoicomonas* between these sea urchin species could be linked to the migratory behavior of *T. ventricosus* because *E. lucunter* does not migrate to the seagrass biotope [29]. *Endozoicomonas* are symbiotic organisms that interact mutually with a variety of marine animals. They can be found in oceans worldwide, although they are more common in slightly temperate and warm tropical waters [73]. They are frequently associated with corals, particularly those in shallow waters, while they can also live in deepwater corals by settling in the soft epithelial tissue [74,75]. Additionally, this taxon has been related to several invertebrates, including sponges, tunicates, sea slugs, and various mollusks [76,77]. *Endozoicomonas* acts as an indicator of the overall health of corals and the species that inhabit coral reefs, minimizing the prevalence of other pathogenic bacteria, and relates to the overall health of corals [78,79]. Functions associated with *Endozoicomonas* include the synthesis of amino acids and vitamins, participating in the nitrogen and sulfur cycles [80], and the transfer of organic molecules that actively support the nutrition of their host [81]. Nevertheless, their precise role and the way in which they affect microorganisms are still unknown.

The genus *Kistimonas* was significantly more abundant in *D. antillarum* when compared to the other sea urchin species from this study. This Proteobacterial taxon was first isolated from the skin of the starfish *Asteria samurensis* [27]. *Kistimonas* is a recently identified lineage of bacteria associated with many marine invertebrates [79,82]. Although Kistimonas symbiotic roles are unknown, their prevalence in *D. antillarum* and other invertebrates may indicate they are a common taxon in these marine invertebrates. *Prolixibacter*, a bacterium found abundant in *E. lucunter*, was originally identified from marine sediment samples [83]. Since this bacterium has been linked with marine sediments, this might explain why it is found in the epibiome of *E. lucunter*. *L. varietagus* had a significantly higher abundance of *Propionigenum* in comparison with all three other sea urchin species evaluated in this study. *Propionigenum* uses malate, aspartate, oxaloacetate, pyruvate, succinate, and fumarate for growth through fermentation producing propionate, acetate, and carbon dioxide (CO_2_) as products [84]. This bacterium thrives in both freshwater and saltwater and in anoxic environments [85,86]. Due to its complex metabolism supplying molecules to its surroundings and its exclusivity in *L. varietagus*, we hypothesize that *Propionigenum* might have a mutualistic but not exclusive relationship with this sea urchin species.

As in our data where we found a direct reflection of the biotope bacterial communities in these Caribbean Sea urchins, preliminary data in other species showed that these bacteria reflect the niches, and when in captivity, sea urchins lose their epibiotic microbial communities. This is important as they are vectors for the transportation of the bacteria in marine ecosystems, thus impacting the ecological and metabolic networks. Indeed, another study on starfish found a pathogen as part of the epibiome and discussed the risks of transmission of epibiotic bacteria transported by echinoderms [87].

We did not find remarkable differences locally among our three sites, likely due to the small geographic distance. The three collection sites were located on the northeastern coast of Puerto Rico and displayed similarities in physicochemical parameters (temperature, salinity, and pH). For example, samples collected in Cataño displayed a lower dominance of Proteobacteria and a higher relative abundance of Firmicutes. This phylum has been found to be associated with oligotrophic environments due to the existence of a phosphate uptake system [87]. However, Cataño, which is located at the entrance of the San Juan Bay, exhibited a moderately higher number of microbial species, which could indicate, along with higher abundances of Bacteroidetes, that this site might be under more anthropogenic impacts.

## 5. Conclusions

This study is a pioneer in characterizing the composition and abundance of the external microbiota of four wild-caught echinoids using NextGen sequencing in the Caribbean region. Species identity rather than biotopes explained the differences found among microbiota profiles, with certain commonalities between those sharing the same habitat (niche). The analyses of these epibionts from distinct species allowed us to determine that each trophic niche impacts the microbial composition of the animal’s spine, which has a greater impact than geographical location, especially regarding reef species. Our findings provide the first report on the external microbial diversity of four Caribbean sea urchin species that could help unravel the biological functions of these epibiotic microbiotas in the host immunological response, providing a unique resource as a reference for the recent die-off.

The microbial profiles of four healthy Caribbean sea urchin species will leverage new efforts in characterizing the *Diadema* die-off. The Caribbean basin is a hotspot for marine pathogenesis due to the presence of over 40 diseases of marine taxa [88]. It is projected that if climate change increases, there will be a rise in the number of new marine ailments, further complicating the situation. Unfortunately, because the etiologies of most marine diseases are still unknown, we are less capable of anticipating and stopping future outbreaks, hindering the development of immediate and long-term solutions. Altogether, these results will improve the understanding of the role microbes play as modulators of population density and community assemblages impacting Caribbean sea urchins. We acknowledge that sea urchins display different seasonal growths, with new parts growing at different seasons, therefore we consider it essential to develop further studies to focus on examining spatial and temporal changes in microbiota along the sea urchin spines and between seasons due to the growth rate patterns [89].

## Figures and Tables

**Table 1:** Sample collection per animal species, with numbers of reads and OTUs by sites. Three samples were removed due to the low number of reads.

Site/Species	Sample Size	Σ Reads	Σ OTU’s
Cataño			
*Diadema antillarum*	6	168,753	1332
*Echinometra lucunter*	6	243,333	2645
*Lytechinus variegatus*	3	10,116	844
*Tripneustes ventricosus*	6	210,389	2369
Total	21	632,591	7190
Cerro Gordo			
*Diadema antillarum*	6	133,216	1640
*Echinometra lucunter*	6	159,781	1443
*Lytechinus variegatus*	1	1,421	197
*Tripneustes ventricosus*	6	105,271	1230
Total	19	399,689	4510
Luquillo			
*Diadema antillarum*	6	61,529	1096
*Echinometra lucunter*	5	128,920	1565
*Tripneustes ventricosus*	6	135,881	1113
Total	17	326,330	3774
Grand Total	57	1,380,601	15,474

**Table 2:** Numbers of Operational Taxonomic Units and read sequences of four sea urchin species at three sites in Puerto Rico.

Species	Sample Size	Σ Reads	Σ OTU’s
*Diadema antillarum*	18	20,194.33 ± 18,481.94	226 ± 147.929236
*Echinometra lucunter*	17	31,296.12 ± 25,474.61	332.53 ± 180.55
*Tripneustes ventricosus*	18	25,085.61 ± 19,937.05	261.78 ± 194.87
*Lytechinus variegatus*	4	2884.25 ± 2241.36	260.25 ± 56.08
Grand Total	57	23,835.26 ± 21,063.80	271.47 ± 173.27

## Data Availability

The 16S-rRNA reads were redeposited in QIITA [44] Bioproject ID 12668, and the raw sequences are available in the European Nucleotide Archive ENA Project: PRJEB40117; ERP123720.

## Supplementary Materials

The supporting supplementary tables can be downloaded at: https://www.mdpi.com/article/10.3390/microorganisms11020391/s1.
