# Supplementary material for: The Epibiotic Microbiota of Wild Caribbean Sea Urchin Spines Is Species Specific
Source: Microorganisms. 2023 Feb 3;11(2):391. doi: 10.3390/microorganisms11020391 (PMC9966300; doi:10.3390/microorganisms11020391)
Supplement: Supplementary file 1 [file microorganisms-11-00391-s001.zip › Suppl Table 1_Abiotic parameters_Sea urchin test microbiota.pdf]

Table S1. Local water physicochemical conditions at three sites of the northeastern coast in Puerto Rico.

**Sites/Parameters**	**Temp (°C)**	**Salinity (o/oo)**	**pH**
Cerro Gordo (CG)	25.6 ± 0.00	33.32 ± 0.04	8.45 ± 0.05
Cataño (IC)	26.08 ± 0.08	33.56 ± 0.05	8.41 ± 0.08
Luquillo (MA)	26.76 ± 0.07	33.8 ± 0.16	8.32 ± 0.05
